# Efficacy and Safety Concerns with Sn-Mesoporphyrin as an Adjunct Therapy in Neonatal Hyperbilirubinemia: A Literature Review

**DOI:** 10.1155/2022/2549161

**Published:** 2022-07-18

**Authors:** Prakar Poudel, Sudhir Adhikari

**Affiliations:** ^1^Devghat Clinic, Chitwan Medical College, Chitwan 44200, Nepal; ^2^Department of Pediatrics, Chitwan Medical College, Chitwan 44200, Nepal

## Abstract

Neonatal hyperbilirubinemia is a frequently observed clinical situation that, sometimes, may result in complications ranging from mild neurodevelopment impairment to serious outcome of kernicterus. The rationale logic of heme oxygenase enzyme inhibition to lower bilirubin levels is intriguing. In compliance with that rationale, metalloporphyrin was discovered. After successful results in in-vitro and animal studies, tin mesoporphyrin is now under phase II clinical trial to test for preventive and therapeutic efficacy in unconjugated hyperbilirubinemia. This review evaluates in-vitro studies, animal studies, and clinical trials for the efficacy and safety of tin analogues of metalloporphyrin. Few alternatives to metalloporphyrins are also available, synchronizing with the same rationale logic of inhibition of bilirubin production, which need further research.

## 1. Introduction

Neonatal jaundice is medically known as neonatal hyperbilirubinemia, in which the bilirubin level of blood raises above five milligrams per deciliter (mg/dL) with yellowish discoloration of the eyes and skin of newborns. This increment of bilirubin level in blood could be due to physiological or pathological causes. Irrespective of etiology, the sequelae of hyperbilirubinemia might range from acute bilirubin encephalopathy to long-term neurodevelopmental impairments with hearing loss, seizure disorders, or cerebral palsy. There is a high chance that kernicterus would occur in neonates with a bilirubin level above 20 mg/dL. With documented multiple case reports, it is estimated that kernicterus, although infrequent, has at least ten percent mortality and at least 70% long-term morbidity [1].

**Figure 1:**
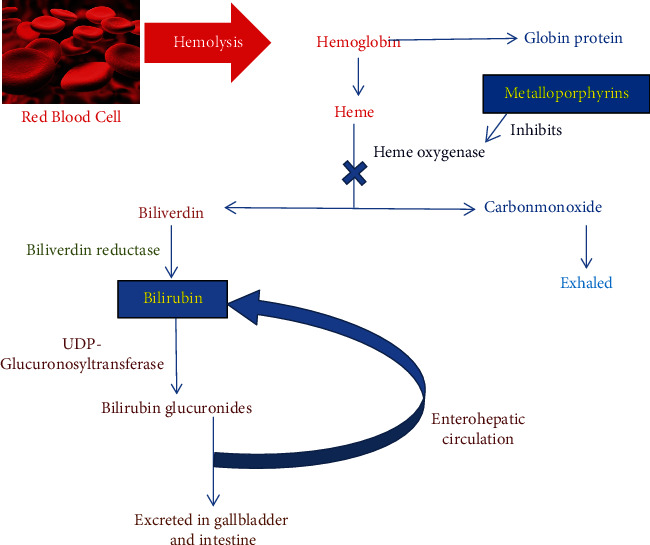
Breakdown of red blood cell. Heme oxygenase enzyme reacts on heme to form biliverdin and eventually bilirubin.

It is well known that heme oxygenase (EC 1·14·14·18) is a rate-limiting microsomal enzyme in the heme degradation pathway and cleaves *α*-methane bridge of heme component in hemoglobin of red blood cells, yielding carbon monoxide (CO), iron ion (Fe^+2^), and biliverdin in equimolar quantities, which is eventually reduced to bilirubin by bilirubin reductase as illustrated in Figure 1. In a mammalian body, heme oxygenase (HO) is highly active in the liver and spleen among all tissues [2].

Initially, Drummond and Kappas observed the existence of metal-metal interactions altering the induction of HO synthesis in their laboratory of The Rockefeller University of New York in 1979 AD [3, 4]. Later, they were fascinated with the results of certain synthetic metalloporphyrins which could act as a competitive substrate for heme in HO reaction and thereby alter cytochrome P-450 dependent oxidative reaction [5].

**Figure 2:**
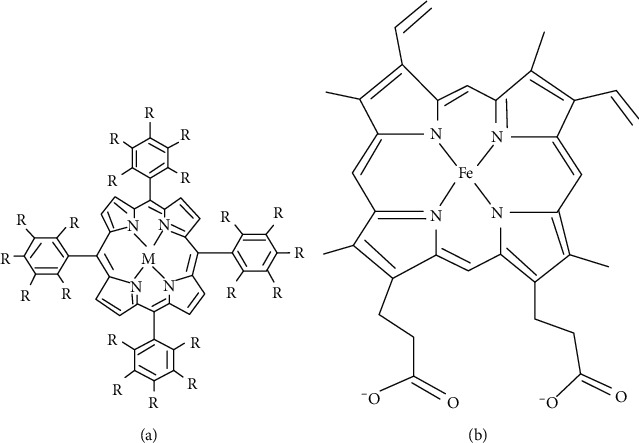
(a) Metalloporphyrin with central symbol “M” for any metal. (b) Heme with central symbol “Fe” for iron.

Synthetic metalloporphyrin, similar to natural heme, consists of ring structured protoporphyrin IX macrocycle with any other metals; tin (Sn), zinc (Zn), manganese (Mn), nickel (Ni), magnesium (Mg), copper (Cu), cadmium (Cd), cobalt (Co), replacing central iron (Fe) of natural heme as illustrated in Figure 2. These synthetic metalloporphyrins, not being able to bind molecular oxygen, cannot be degraded by HO enzyme, unlike heme. Hence, in the presence of these metalloporphyrins, heme cannot be degraded into biliverdin and eventually bilirubin. Among those heme analogues which they have extensively worked with, Sn-protoporphyrin proved to be a strong competitive inhibitor for heme in HO redox reaction [6–9].

Theoretically, a strategy to inhibit the production of bilirubin seems intriguing to treat hyperbilirubinemia. Since the early 1980s, scientists had extensively worked with synthetic metalloporphyrins, which coincides with that rationale logic [6, 7, 8]. The scientists formulated certain criteria for the identification of a suitable metalloporphyrin for the treatment of neonatal hyperbilirubinemia. The ideal inhibitor (a) contains a metal that occurs naturally in the body or is harmless in trace amounts, (b) is not degraded in tissue to possibly harmful substances, (c) inhibits heme oxygenase effectively at relatively low doses, and (d) does not participate in photodestructive reactions [10].

In current medical practice, the therapeutic measures for neonatal hyperbilirubinemia in hospitals are phototherapy, exchange blood transfusion, and, occasionally, intravenous immunoglobulins. The discharge of a term newborn is individualized considering medical, social, and economic situation of each case. Meanwhile, the American Academy of Pediatrics suggests a short hospital stay of less than 48 hours after vaginal birth and less than 96 hours after cesarean delivery for healthy term newborns [11]. However, for some obscure cases, this recommendation may seem to be an early discharge from the hospital, thereby readmission for neonatal hyperbilirubinemia and the need for phototherapy.

With low evidence on efficacy and safety in human neonates, synthetic metalloporphyrins are still not licensed for routine clinical use in countries like USA or UK. However, now, tin mesoporphyrin (Sn-mesoporphyrin: Sn-MP) is under a phase II clinical trial to compare preventive and therapeutic efficacy in unconjugated hyperbilirubinemia [12]. Therefore, this review entails literature pieces of evidence on tin (Sn) analogues of metalloporphyrin and its modified forms, in regard to their efficacy, potency, and safety, for prophylactic and therapeutic use in neonatal hyperbilirubinemia.

## 2. Materials and Methods

For the identification of records in PubMed (MEDLINE), the following MeSH search strategy was used in “query box”: (“Metalloporphyrins”[Mesh]) AND “Jaundice, Neonatal”[Mesh]. Articles were selected from all 62 records that were relevant to the topic of this review. Records were also identified in Google scholar database with search terms and Boolean operators: (“jaundice” OR “hyperbilirubinemia”) AND (“mesoporphyrin”). Reports were included from the first 50 articles based on relevance of the review topic. After searching with the term “mesoporphyrin AND hyperbilirubinemia” in the scholarly works section of LENS database, all 70 articles were selectively screened. Additionally, for the search in the online repository ClinicalTrials.gov, “Neonatal Hyperbilirubinemia” term was used in “Condition or disease” box and “Mesoporphyrin” in “Other terms” box. A total of seven clinical trials were found. Only papers published in English language were selected. Further relevant articles were also included from the reference section of selected articles with available full text.

## 3. Results

The reports that favor efficacy and concern the safety of Sn-mesoporphyrin are listed in Table 1.

## 4. Discussion

### 4.1. In Vitro and Animal Studies with Sn Analogue Metalloporphyrin

Tin analogue of metalloporphyrin is an emerging interest in the scientific community. Both in-vitro studies and animal models revealed that tin protoporphyrin (Sn-protoporphyrin; Sn-PP) could inhibit both liver and spleen HO enzyme activities competitively and potently within 24 hours of interaction and thereby block in-vivo production of bilirubin by 24 hours after birth [6, 7, 13]. Additionally, the concern of accumulated heme in tissue following Sn-PP administration was also addressed with the finding of stimulated excretion of heme into the bile [14].

In due course of time, the study demonstrated dose-dependent effect of Sn-PP on HO inhibition. However, it also further revealed the inability of Sn-PP to entirely decrease plasma bilirubin levels to a normal level, except the rapid decline observed shortly after initial administration in an intensive repeated dose treatment. Instead, the effect was observed to be sustained and prolonged which could be explained by the subcutaneous route of administration used in the study [15]. However, a safety concern still lingered after two deaths in a study of Sn-PP in primates. The scientists of that study explained those deaths with the suspected reasons of high level of single dose or drug sensitivity, which does not equate with multiple previous animal studies [16]. A study also discovered that Sn-PP can traverse both the placental barrier and blood-brain barrier of neonates, inhibiting tissue HO activity in fetus and brain HO in neonates respectively, and thereby following such prenatal administration, suppresses postnatal hyperbilirubinemia [17].

Sn-mesoporphyrin (Sn-MP) is a modified molecule of Sn-PP in which vinyl groups at C2 and C4 have been reduced to ethyl groups. A comparative study of the potency between these two molecules demonstrated that the effectiveness of Sn-MP is tenfold or more compared with Sn-PP to inhibit HO activity [18]. In addition, a treatment model of a pathological disease, Dubin Johnson syndrome, in an animal study demonstrated marked inhibition of HO activity in the liver and spleen, but not in the brain. The weekly injections of Sn-MP significantly resulted in a low plasma bilirubin concentration for extended periods [19]. Few studies also showed that even an oral administration of Sn-MP resulted in a persistent 24 hours decline of plasma bilirubin level [20, 21]. Another study further demonstrated that repeated heme exposure, a condition similar to any hemolytic disease pathology, will require a higher dose of Sn-MP in the neonate to inhibit both liver and brain HO activity in a long term [22]. In conclusion, a neonate with hemolytic pathology will require a higher dose of Sn-MP to observe a significant and prolonged decline in bilirubin levels.

Despite the beneficial efficacy of metalloporphyrins, the photosensitizing property eludes clinical use and questions their safety concerns. A study pointed out that Sn-MP is a fatal and potential phototoxic substance in neonates with dose-dependent mortality [23]. In fact, all tin analogues (Sn-PP, Sn-MP, Sn-deuteroporphyrin, and Sn-bisglycol deuteroporphyrin) were powerful in-vitro photooxidizers capable of producing singlet oxygen, which could oxidize a large number of organic molecules including bilirubin [24–26]. This oxidation reaction inside the body of neonates could be the reason for their death [23]. In the other hand, the chromium analogue was considered an ideal HO inhibitor without photoreactive properties. However, similar to Sn-MP, chromium mesoporphyrin was found to be impermeable to blood-brain barrier contrary to the property of Sn-PP [10, 27, 28]. These properties make the chromium analogues more suitable and alternative to the tin analogue of mesoporphyrin for the control of neonatal hyperbilirubinemia and deserve further research.

It is known that heme oxygenase enzyme system exists as isoforms (HO-1 and HO-2). These isoforms are transcribed from independent genes and there is no resemblance at gene structure, regulation, or tissue expression patterns. Despite these differences, both HO-1 and HO-2 catalyze the same reaction of heme degradation [29]. However, Wong and Pierce along with their team demonstrated that all metalloporphyrins selectively inhibit HO-2 more than HO-1 isoform, except zinc metalloporphyrin [30, 31]. Hence, further studies are also needed to find an ideal form of metalloporphyrin that can selectively inhibit the inducible HO-1 isoform, without affecting the constitutive activity of HO-2 isoform.

### 4.2. Human Studies with Sn-Mesoporphyrin

In clinical practice, it is also not uncommon for physicians to come across neonates of parents who are Jehovah's Witness adherents, community members to reject the use of blood for exchange transfusion. Consequently, a physician, in compliance with the beneficence principle of medical ethics, may be obliged to initiate legal action to impel an exchange transfusion procedure for refractory hyperbilirubinemia. The outcomes following the adjunct use of Sn-MP with phototherapy in two case reports with such legal circumstances dictated the ethical demand in clinical practice [32]. Along with that, Sn-MP was also found to be effective in cases of Crigler-Najjar disease [33, 34].

Following the promising results with the tin analogues of metalloporphyrin in both in vitro and animal studies, researchers also found favorable outcomes with randomized control trials (RCT) in human neonates. Multiple RCTs depicted that an early single-dose administration of Sn-MP in both preterm and term neonates significantly reduces total plasma bilirubin concentration following treatment.

In contrast to an animal study [15], human neonatal study demonstrated that escalating doses may not necessarily decrease total plasma bilirubin levels in dose-dependent manner [35, 36]. Rather, a prolonged sustained low level of bilirubin was observed, similar to the animal study [15], although still significantly lower than the control group [35, 37]. This inferred that the profound inhibitory effect of Sn-MP is only evident by 24 to 48 hours with the initial sharp decline [38], but significant difference in bilirubin levels compared to the control group occurs only at three to five days posttreatment. Hence, further study with intravenous administration is also required to avoid the confounding effect of the route of administration in finding the exact efficacy of Sn-MP.

Furthermore, a single adjunct dose of Sn-MP also resulted in a marked reduction of both the need and duration of phototherapy or even elimination of the need for phototherapy [35–37, 39–41]. Additionally, Sn-MP also benefited as a prophylactic therapy in glucose-6-phosphate dehydrogenase- (G6PD-) deficient neonates, sustaining low bilirubin levels and lowering the need and duration for phototherapy [42, 43].

### 4.3. Recent Clinical Trials with Sn-Mesoporphyrin

To this date, total seven clinical trials are listed in the online repository; with one being no longer available. Among those, two clinical trials posted their results [44].

The results of one clinical trial, with a focus on the safety and efficacy of Sn-MP, revealed that escalating intramuscular dose administration of Sn-MP might not bring an expected proportional decrease in adjusted total serum bilirubin within 48 hours after treatment. This analysis was made because the primary outcome was not statistically significant except for the lowest dose of 1.5 milligram/kilogram body weight (mg/kg). However, attrition bias could also be a reason for this result, not to exclude a combined effect of confounders: blood circulation, infections, and fever. Additionally, the results also showed that only 4.5 mg/kg dose administration brought a significant decrease in total plasma bilirubin level measured at 48 hours posttreatment. Surprisingly, this trial depicted low efficacy of Sn-MP. In terms of safety, a maximum of 4.5 mg/kg intramuscular dose did not cause mortality despite a high incidence of non-serious adverse events (skin erythema) compared to the control group. Apart from a 13.33% absolute risk reduction for hyperbilirubinemia, Sn-MP could not reduce the risk for other serious events (anemia, meningitis) [45].

Another clinical trial demonstrated that a single intramuscular dose (3 mg/kg or 4.5 mg/kg) adjunct with phototherapy could decrease total serum bilirubin levels at 48 hours after treatment with statistical significance. Similarly, there was no associated mortality, but the group receiving 4.5 mg/kg dose was at 3.23% risk of developing infection and sepsis compared to both lower dose and placebo groups. Neonates in the treatment group markedly showed some non-serious adverse effects: increased reticulocyte count, increased aspartate aminotransferase, erythema and maculopapular rash [46].

### 4.4. Alternatives for Further Research

Few alternatives can also be studied with the same rationale logic of inhibition of HO. The use of a lyophilized liposomal Sn-MP formulations, being five to 20 times higher effective than aqueous Sn-MP, can also bring targeted delivery to the spleen for HO inhibition, thereby requiring a lower dose and fewer possible side effects [47]. Halogenized form of tin analogue like Sn^+4^-diiododeuteroporphyrin can also be another option to address the phototoxicity issue, retaining similar potent inhibition [48]. Further studies on the relationship of phototoxicity of metalloporphyrins with the emission spectra of light used in phototherapy can also bring breakthrough discoveries in this field. Chromium analogue of mesoporphyrin is another metalloporphyrin to study. A structurally different compound like imidazole-dioxolanes, with comparatively high selection for inducible HO-1 isozyme, could also be an alternative to metalloporphyrins which need further research as well [49, 50]. Therefore, a cost-effective and benefit analysis comparing metalloporphyrins with alternative compounds would be helpful, rather than heedlessly spending in research with metalloporphyrins.

This review was formulated on available pieces of evidence, but the quality assessment was not performed for selected studies. Studies included were also mixed with different study designs.

## 5. Conclusions

In regard to the significant results in multiple clinical trials, there is minimal doubt on the efficacy of Sn-MP. However, the next question is to find its comparative efficiency as prophylactics or therapeutics. With mixed and inadequate evidence on both short- and long-term safety, it can be a quick decision for its clinical use in neonatal hyperbilirubinemia. Now, the results of forthcoming clinical trials can shed further light on their safety concerns.

## Figures and Tables

**Table 1:** Reports for and against Sn-mesoporphyrin in neonatal hyperbilirubinemia.

Reports favoring efficacy	Reports with safety concerns
Drummond et al. [18]	Hintz et al. [23]
Drummond and Kappas [19]	
DeSandre et al. [20]	Fort and Gold [24]
Morioka et al. [21]	
Wong et al. [22]	Vreman et al. [25]
Rosenfeld et al. [35]	
Rosenfeld et al. [36]	
Bhutani et al. [37]	
Maisels and Yang [38]	
Valaes et al. [39]	
Kappas et al. [40]	
Martinez et al. [41]	
Valaes et al. [42]	
Kappas et al. [43]	
Case reports:	
Kappas et al. [32]	
Rubaltelli et al. [33]	
Galbraith et al. [34]	
Maisels and Yang [38]	

## Data Availability

The data used to support the findings of this study are included within the article. To access clinical trials in online repository, link is as follows: https://clinicaltrials.gov/ct2/results?cond=Neonatal+Hyperbilirubinemia&term=Mesoporphyrin&cntry=&state=&city=&dist=

## References

[1] Ip S., Chung M., Kulig J. (2004). An evidence-based review of important issues concerning neonatal hyperbilirubinemia. *Pediatrics*.

[2] Tenhunen R., Marver H. S., Schmid R. (1969). Microsomal Heme Oxygenase: CHARACTERIZATION OF THE ENZYME. *The Journal of Biological Chemistry*.

[3] Drummond G. S., Kappas A. (1979). Manganese and zinc blockade of enzyme induction: studies with microsomal heme oxygenase. *Proceedings of the National Academy of Sciences*.

[4] Drummond G. S., Kappas A. (1980). Metal ion interactions in the control of heme oxygenase induction in liver and kidney. *The Biochemical Journal*.

[5] Maines M. D., Ibrahim N. G., Kappas A. (1977). Solubilization and partial purification of heme oxygenase from rat liver. *The Journal of Biological Chemistry*.

[6] Drummond G. S., Kappas A. (1981). Prevention of neonatal hyperbilirubinemia by tin protoporphyrin IX, a potent competitive inhibitor of heme oxidation. *Proc Natl Acad Sci USA*.

[7] Drummond G. S., Kappas A. (1982). Chemoprevention of neonatal jaundice: potency of tin-protoporphyrin in an animal model. *Science*.

[8] Yoshinaga T., Sassa S., Kappas A. (1982). Purification and properties of bovine spleen heme oxygenase. Amino acid composition and sites of action of inhibitors of heme oxidation. *The Journal of Biological Chemistry*.

[9] Kappas A., Drummond G. S. (1986). Control of heme metabolism with synthetic metalloporphyrins. *The Journal of Clinical Investigation*.

[10] Verman H. J., Ekstrand B. C., Stevenson D. K. (1993). Selection of metalloporphyrin heme oxygenase inhibitors based on potency and photoreactivity. *Pediatric Research*.

[11] Committee on Fetus and Newborn (2010). Hospital stay for healthy term newborns. *Pediatrics*.

[12] Kappas A. (1999). Phase II randomized study of tin mesoporphyrin for neonatal hyperbilirubinemia. *Clinical Trials.gov*.

[13] Drummond G. S., Kappas A. (1984). An experimental model of postnatal jaundice in the suckling rat. Suppression of induced hyperbilirubinemia by Sn-protoporphyrin. *The Journal of Clinical Investigation*.

[14] Kappas A., Simionatto C. S., Drummond G. S., Sassa S., Anderson K. E. (1985). The liver excretes large amounts of heme into bile when heme oxygenase is inhibited competitively by Sn-protoporphyrin. *Proceedings of the National Academy of Sciences*.

[15] Sassa S., Drummond G. S., Bernstein S., Kappas A. (1983). Tin-protoporphyrin suppression of hyperbilirubinemia in mutant mice with severe hemolytic anemia. *Blood*.

[16] Cornelius C. E., Rodgers P. A. (1984). Prevention of neonatal hyperbilirubinemia in rhesus monkeys by tin-protoporphyrin. *Pediatric Research*.

[17] Drummond G. S., Kappas A. (1986). Sn-protoporphyrin inhibition of fetal and neonatal brain heme oxygenase. Transplacental passage of the metalloporphyrin and prenatal suppression of hyperbilirubinemia in the newborn animal. *The Journal of Clinical Investigation*.

[18] Drummond G. S., Galbraith R. A., Sardana M. K., Kappas A. (1987). Reduction of the C2 and C4 vinyl groups of Sn-protoporphyrin to form Sn-mesoporphyrin markedly enhances the ability of the metalloporphyrin to inhibit in vivo heme catabolism. *Archives of Biochemistry and Biophysics*.

[19] Drummond G. S., Kappas A. (1998). Sn-mesoporphyrin suppression of hyperbilirubinemia in EHBR/Eis rats, an animal model of Dubin-Johnson syndrome. *Pharmacology*.

[20] DeSandre G. H., Wong R. J., Morioka I., Contag C. H., Stevenson D. K. (2006). The effectiveness of oral tin mesoporphyrin prophylaxis in reducing bilirubin production after an oral heme load in a transgenic mouse model. *Biology of the Neonate*.

[21] Morioka I., Wong R. J., Abate A., Vreman H. J., Contag C. H., Stevenson D. K. (2006). Systemic effects of orally-administered zinc and tin (IV) metalloporphyrins on heme oxygenase expression in mice. *Pediatric Research*.

[22] Wong R. J., Morioka I., Muchova L., Vreman H. J., Stevenson D. K. (2007). 73 inhibition of heme oxygenase activity following repeated heme loads by tin mesoporphyrin in newborn mice. *Journal of Investigative Medicine*.

[23] Hintz S. R., Vreman H. J., Stevenson D. K. (2019). Mortality of metalloporphyrin-treated neonatal rats after light exposure. *Developmental Pharmacology and Therapeutics*.

[24] Fort F. L., Gold J. (1989). Phototoxicity of tin protoporphyrin, tin mesoporphyrin and tin diiododeuteroporphyrin under neonatal phototherapy conditions. *Pediatrics*.

[25] Vreman H. J., Gillman M. J., Downum K. R., Stevenson D. K. (2017). In vitro generation of carbon monoxide from organic molecules and synthetic metalloporphyrins mediated by light. *Developmental Pharmacology and Therapeutics*.

[26] Keino H., Nagae H., Mimura S., Watanabe K., Kashiwamata S. (1990). Dangerous effects of tin-protoporphyrin plus photoirradiation on neonatal rats. *European Journal of Pediatrics*.

[27] Vreman H. J., Cipkala D. A., Stevenson D. K. (1996). Characterization of porphyrin heme oxygenase inhibitors. *Canadian Journal of Physiology and Pharmacology*.

[28] Schulz S., Wong R. J., Kalish F. S. (2012). Effect of light exposure on metalloporphyrin-treated newborn mice. *Pediatric Research*.

[29] Maines M. D. (1997). The heme oxygenase system: a regulator of second messenger gases. *Annual Review of Pharmacology and Toxicology*.

[30] Wong R. J., Vreman H. J., Schulz S., Kalish F. S., Pierce N. W., Stevenson D. K. (2011). In vitro inhibition of heme oxygenase isoenzymes by metalloporphyrins. *Journal of Perinatology*.

[31] Pierce N. W., Wong R. J., Morioka I., Vreman H. J., Stevenson D. K. (2006). 238 metalloporphyrin inhibition of in vitro mouse heme oxygenase isozyme activity. *Journal of Investigative Medicine*.

[32] Kappas A., Drummond G. S., Munson D. P., Marshall J. R. (2001). Sn-Mesoporphyrin interdiction of severe hyperbilirubinemia in Jehovah's Witness newborns as an alternative to exchange transfusion. *Pediatrics*.

[33] Rubaltelli F. F., Dario C., Zancan L. (1995). Congenital nonobstructive, nonhemolytic jaundice: effect of tin-mesoporphyrin. *Pediatrics*.

[34] Galbraith R. A., Drummond G. S., Kappas A. (1992). Suppression of bilirubin production in the Crigler-Najjar type I syndrome: studies with the heme oxygenase inhibitor tin-mesoporphyrin. *Pediatrics*.

[35] Rosenfeld W. N., Hudak M. L., Ruiz N. (2022). Stannsoporfin with phototherapy to treat hyperbilirubinemia in newborn hemolytic disease. *Journal of Perinatology*.

[36] Rosenfeld W. N., Hudak M. L., Ruiz N. (2019). Stannsoporphin (SnMP), tin mesoporphyrin, combined with phototherapy (PT) is superior to PT alone in neonates with hyperbilirubinemia (HB) and hemolysis. *Pediatrics*.

[37] Bhutani V. K., Poland R., Meloy L. D., Hegyi T., Fanaroff A. A., Maisels M. J. (2016). Clinical trial of tin mesoporphyrin to prevent neonatal hyperbilirubinemia. *Journal of Perinatology*.

[38] Maisels M. J., Yang H. (2012). Tin-mesoporphyrin in the treatment of refractory hyperbilirubinemia due to Rh incompatibility. *Journal of Perinatology*.

[39] Valaes T., Petmezaki S., Henschke C., Drummond G. S., Kappas A. (1994). Control of jaundice in preterm newborns by an inhibitor of bilirubin production: studies with tin-mesoporphyrin. *Pediatrics*.

[40] Kappas A., Drummond G. S., Henschke C., Valaes T. (1995). Direct comparison of Sn-mesoporphyrin, an inhibitor of bilirubin production and phototherapy in controlling hyperbilirubinemia in term and near-term newborns. *Pediatrics*.

[41] Martinez J. C., Garcia H. O., Otheguy L. E., Drummond G. S., Kappas A. (1999). Control of severe hyperbilirubinemia in full-term newborns with the inhibitor of bilirubin production Sn-mesoporphyrin. *Pediatrics*.

[42] Valaes T., Drummond G. S., Kappas A. (1998). Control of hyperbilirubinemia in glucose-6-phosphate dehydrogenase-deficient newborns using an inhibitor of bilirubin production, Sn-mesoporphyrin. *Pediatrics*.

[43] Kappas A., Drummond G. S., Valaes T. (2001). A single dose of Sn-mesoporphyrin prevents development of severe hyperbilirubinemia in glucose-6-phosphate dehydrogenase-deficient newborns. *Pediatrics*.

[44] US NLM-NIH (2021). *Clinical Trials.gov*.

[45] Mallinckrodt Clinical Team Leader (2009). A Phase 2b, multicenter, single-dose, blinded, randomized, placebo-controlled, dose-escalation, safety and efficacy trial of stannsoporfin in neonates with hyperbilirubinemia. *Clinical Trials.gov*.

[46] Mallinckrodt Global Clinical Leader (2013). A Phase 2b multicenter, single dose, randomized, double blind, placebo-controlled, parallel-group study evaluating the safety and efficacy of two doses of stannsoporfin in combination with phototherapy in neonates. *Clinical Trials.gov*.

[47] Cannon J. B., Martin C., Drummond G. S., Kappas A. (1993). Targeted delivery of a heme oxygenase inhibitor with a lyophilized liposomal tin mesoporphyrin formulation. *Pharmaceutical Research*.

[48] Drummond G. S., Greenbaum N. L., Kappas A. (1991). Tin (Sn+4)-diiododeuteroporphyrin; an in vitro and in vivo inhibitor of heme oxygenase with substantially reduced photoactive properties. *The Journal of Pharmacology and Experimental Therapeutics*.

[49] Roman G., Riley J. G., Vlahakis J. Z. (2007). Heme oxygenase inhibition by 2-oxy-substituted 1-(1H-imidazol-1-yl)-4-phenylbutanes: effect of halogen substitution in the phenyl ring. *Bioorganic & Medicinal Chemistry*.

[50] Rahman M. N., Vlahakis J. Z., Roman G. (2010). Structural characterization of human heme oxygenase-1 in complex with azole-based inhibitors. *Journal of Inorganic Biochemistry*.

